# The genome sequence of the white-throated dipper, *Cinclus cinclus *(Linnaeus, 1758)

**DOI:** 10.12688/wellcomeopenres.23291.1

**Published:** 2024-11-05

**Authors:** Stuart P. Sharp, Wellcome Sanger Institute Tree of Life Management, Samples and Laboratory team, Wellcome Sanger Institute Scientific Operations: Sequencing Operations, Wellcome Sanger Institute Tree of Life Core Informatics team, Tree of Life Core Informatics collective, Darwin Tree of Life Consortium

**Affiliations:** 1 Lancaster Environment Centre, Lancaster University, Lancaster, England, UK

**Keywords:** Cinclus cinclus, white-throated dipper, genome sequence, chromosomal, Passeriformes

## Abstract

We present a genome assembly from a juvenile male *Cinclus cinclus* (the white-throated dipper; Chordata; Aves; Passeriformes; Cinclidae). The genome sequence has a total length of 1,170.80 megabases. Most of the assembly (93.88%) is scaffolded into 39 chromosomal pseudomolecules, including the Z sex chromosome. The mitochondrial genome has also been assembled and is 18.67 kilobases in length.

## Species taxonomy

Eukaryota; Opisthokonta; Metazoa; Eumetazoa; Bilateria; Deuterostomia; Chordata; Craniata; Vertebrata; Gnathostomata; Teleostomi; Euteleostomi; Sarcopterygii; Dipnotetrapodomorpha; Tetrapoda; Amniota; Sauropsida; Sauria; Archelosauria; Archosauria; Dinosauria; Saurischia; Theropoda; Coelurosauria; Aves; Neognathae; Neoaves; Telluraves; Australaves; Passeriformes; Cinclidae; *Cinclus*; *Cinclus cinclus* (Linnaeus, 1758) (NCBI:txid127875).

## Background

**Figure 1.**
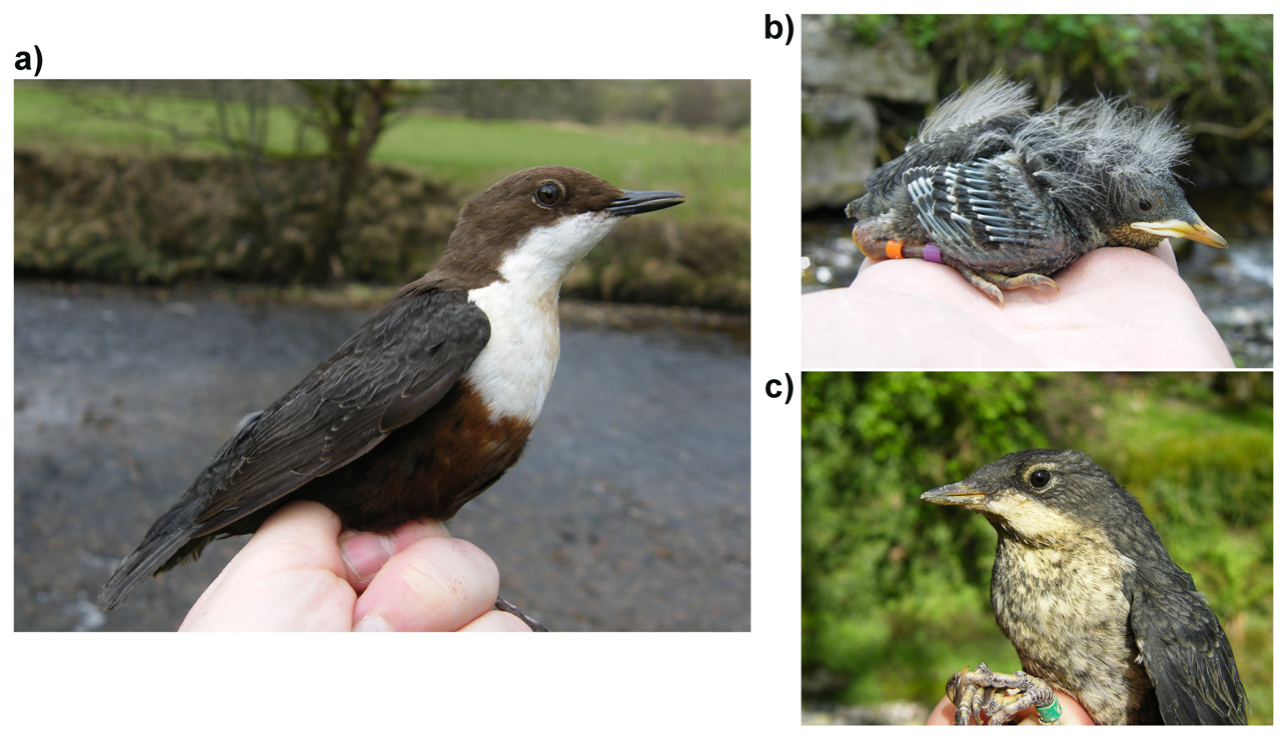
Photographs of *Cinclus cinclus* by Stuart Sharp: **a**) Adult, **b**) 9-day-old nestling and **c**) Juvenile.

The dippers are five species of highly specialised freshwater passerines in the family Cinclidae ( Ormerod, 2005). The most widespread of these is the white-throated dipper ( *Cinclus cinclus*) ( Figure 1), which occurs throughout much of Europe and areas of North Africa, the Middle East, and Central Asia ( Tyler & Ormerod, 1994). A thrush-sized bird with a short tail, the species is easily recognised from its dark brown upperparts and pure white throat and belly, although the plumage varies across its global range and numerous subspecies have been described ( Tyler & Ormerod, 1994). While the white-throated dipper is classified as Least Concern by the IUCN due to its large population size and range ( BirdLife International, 2024), it has undergone significant decline in the UK and is currently amber-listed in the ‘Birds of Conservation Concern’ ( Stanbury *et al.*, 2021).

The white-throated dipper favours upland, fast-flowing rivers and streams, but can be found at lower elevations and even in urban areas where well-oxygenated and unpolluted water with stony substrate provides sufficient prey ( Tyler & Ormerod, 1994). Birds swim and dive to feed predominantly on freshwater invertebrates, notably the larvae and nymphs of caddisflies (Trichoptera), stoneflies (Plecoptera) and mayflies (Ephemeroptera), and build their nests close to the water on rock faces or ledges, in tree roots or mossy banks, or in manmade structures ( Tyler & Ormerod, 1994). This aquatic lifestyle requires a suite of morphological and physiological adaptations, including dense plumage and a large preen gland for waterproofing; high haemoglobin levels and heart rate adjustment associated with diving; and well-developed sphincter muscles in the iris providing powerful accommodation of vision in both air and water ( Ormerod, 2005; Tyler & Ormerod, 1994).

The specialised riverine ecology of this species and its dependence on freshwater macroinvertebrates make the white-throated dipper an excellent bioindicator for the health of riparian ecosystems. For example, breeding density and performance are lower in acidic waters ( Tyler & Ormerod, 1992), and eggs and tissues bioaccumulate various pollutants (e.g. Morrissey *et al.*, 2013) including plastics (e.g. D’Souza *et al.*, 2020). Furthermore, long-term studies of these birds offer an excellent model for understanding how wildlife is affected by climate change (e.g. Nilsson *et al.*, 2011), together with a range of topics in population and behavioural ecology (e.g. Crowther *et al.*, 2018; Magoolagan *et al.*, 2018). Molecular studies of white-throated dippers therefore offer great potential for studying the physiological, behavioural and population-level mechanisms by which this species responds to the many forms of environmental change.

Here we present a chromosomally complete genome sequence for *Cinclus cinclus*, based on a specimen from Sedbergh, Cumbria, United Kingdom.

## Genome sequence report

The genome of a juvenile male *Cinclus cinclus* was sequenced using Pacific Biosciences single-molecule HiFi long reads, generating a total of 49.72 Gb (gigabases) from 4.19 million reads, providing approximately 41-fold coverage, based on the GenomeScope estimate of the genome size. Primary assembly contigs were scaffolded with chromosome conformation Hi-C data, which produced 94.01 Gb from 622.58 million reads. Specimen and sequencing information is summarised in Table 1.

**Table 1.** Specimen and sequencing data for *Cinclus cinclus*.

Project information			
**Study title**	*Cinclus cinclus* (white-throated dipper)		
**Umbrella BioProject**	PRJEB61999		
**BioSample**	SAMEA9679959		
**NCBI taxonomy ID**	127875		
Specimen information			
**Technology**	**ToLID**	**BioSample accession**	**Organism part**
**PacBio long read sequencing**	bCinCin1	SAMEA9679960	blood
**Hi-C sequencing**	bCinCin2	SAMEA9679968	blood
**RNA sequencing**	bCinCin4	SAMEA9679970	blood
Sequencing information			
**Platform**	**Run accession**	**Read count**	**Base count (Gb)**
**Hi-C Illumina NovaSeq 6000**	ERR11439604	6.23e+08	94.01
**PacBio Sequel IIe**	ERR11458787	1.84e+06	22.26
**PacBio Sequel IIe**	ERR11458788	2.35e+06	27.46
**RNA Illumina NovaSeq 6000**	ERR11641138	4.51e+07	6.81

**Figure 2.**
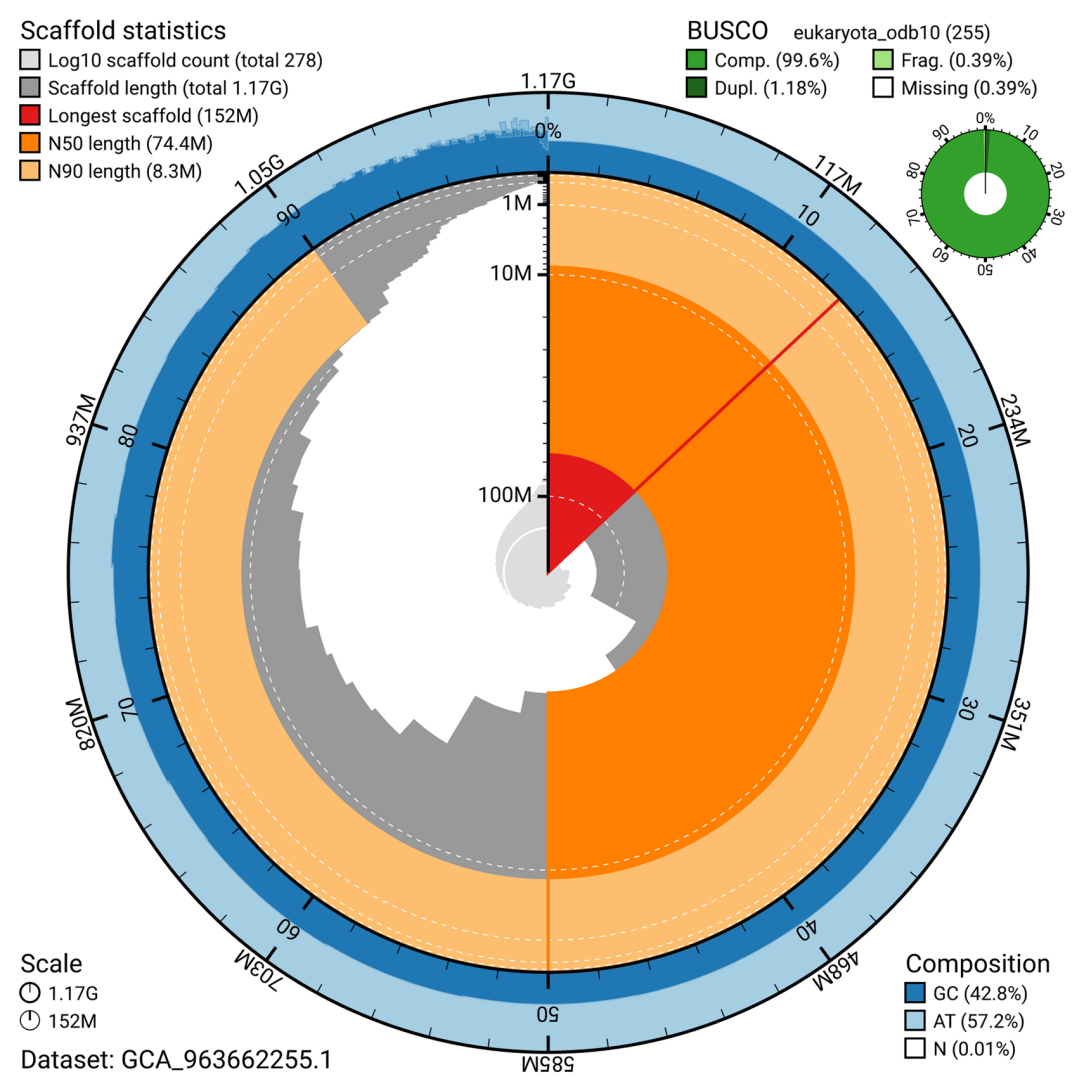
Genome assembly of *Cinclus cinclus*, bCinCin1.1: metrics. The BlobToolKit snail plot shows N50 metrics and BUSCO gene completeness. The main plot is divided into 1,000 size-ordered bins around the circumference with each bin representing 0.1% of the 1,170,855,695 bp assembly. The distribution of scaffold lengths is shown in dark grey with the plot radius scaled to the longest scaffold present in the assembly (152,364,536 bp, shown in red). Orange and pale-orange arcs show the N50 and N90 scaffold lengths (74,357,285 and 8,299,355 bp), respectively. The pale grey spiral shows the cumulative scaffold count on a log scale with white scale lines showing successive orders of magnitude. The blue and pale-blue area around the outside of the plot shows the distribution of GC, AT and N percentages in the same bins as the inner plot. A summary of complete, fragmented, duplicated and missing BUSCO genes in the passeriformes_odb10 set is shown in the top right. An interactive version of this figure is available at https://blobtoolkit.genomehubs.org/view/GCA_963662255.1/dataset/GCA_963662255.1/snail.

**Figure 3.**
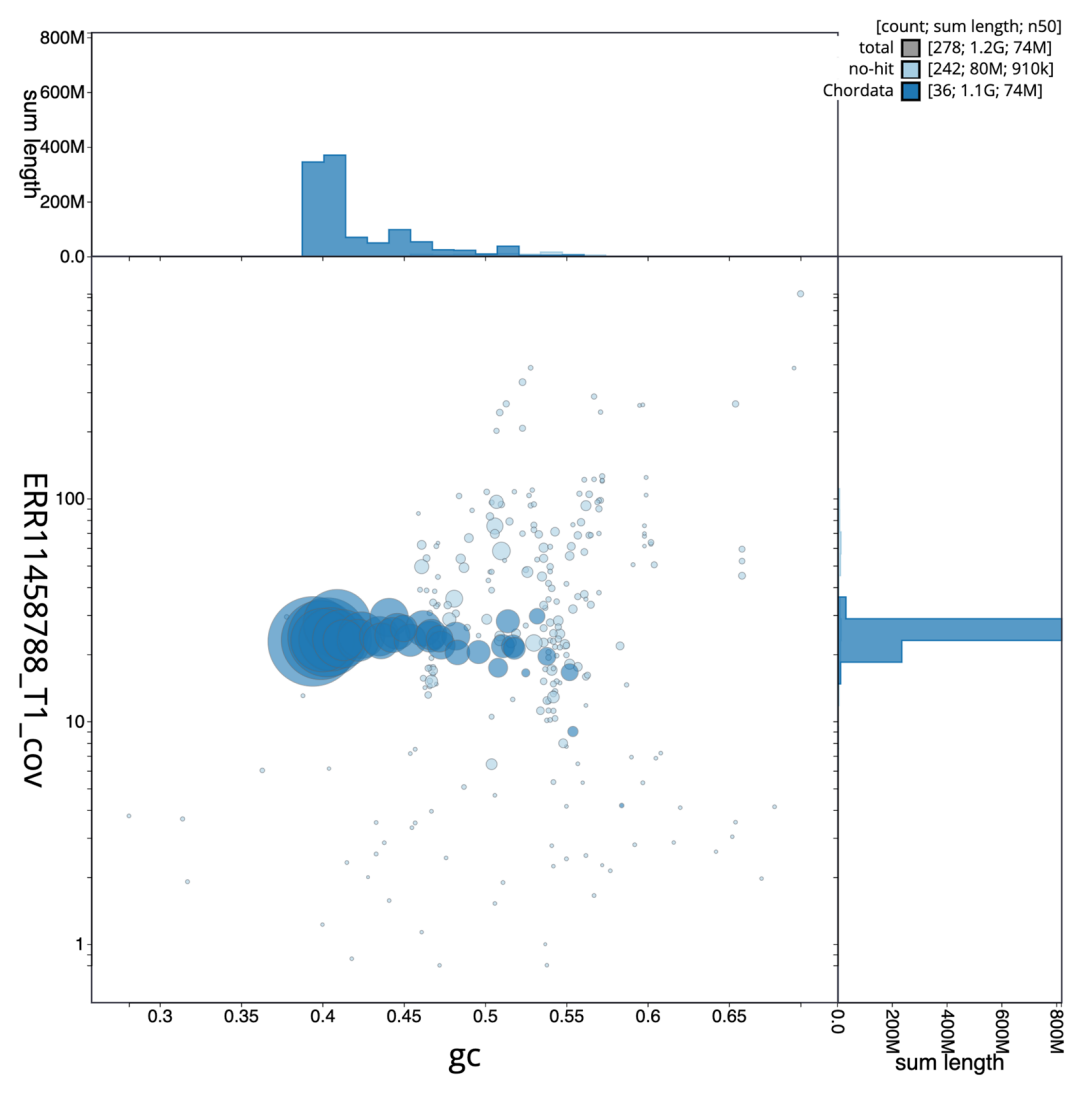
Genome assembly of *Cinclus cinclus* assembly bCinCin1.1: BlobToolKit GC-coverage plot showing sequence coverage (vertical axis) and GC content (horizontal axis). The circles represent scaffolds, with the size proportional to scaffold length and the colour representing phylum membership. The histograms along the axes display the total length of sequences distributed across different levels of coverage and GC content. An interactive version of this figure is available at https://blobtoolkit.genomehubs.org/view/GCA_963662255.1/dataset/GCA_963662255.1/blob.

**Figure 4.**
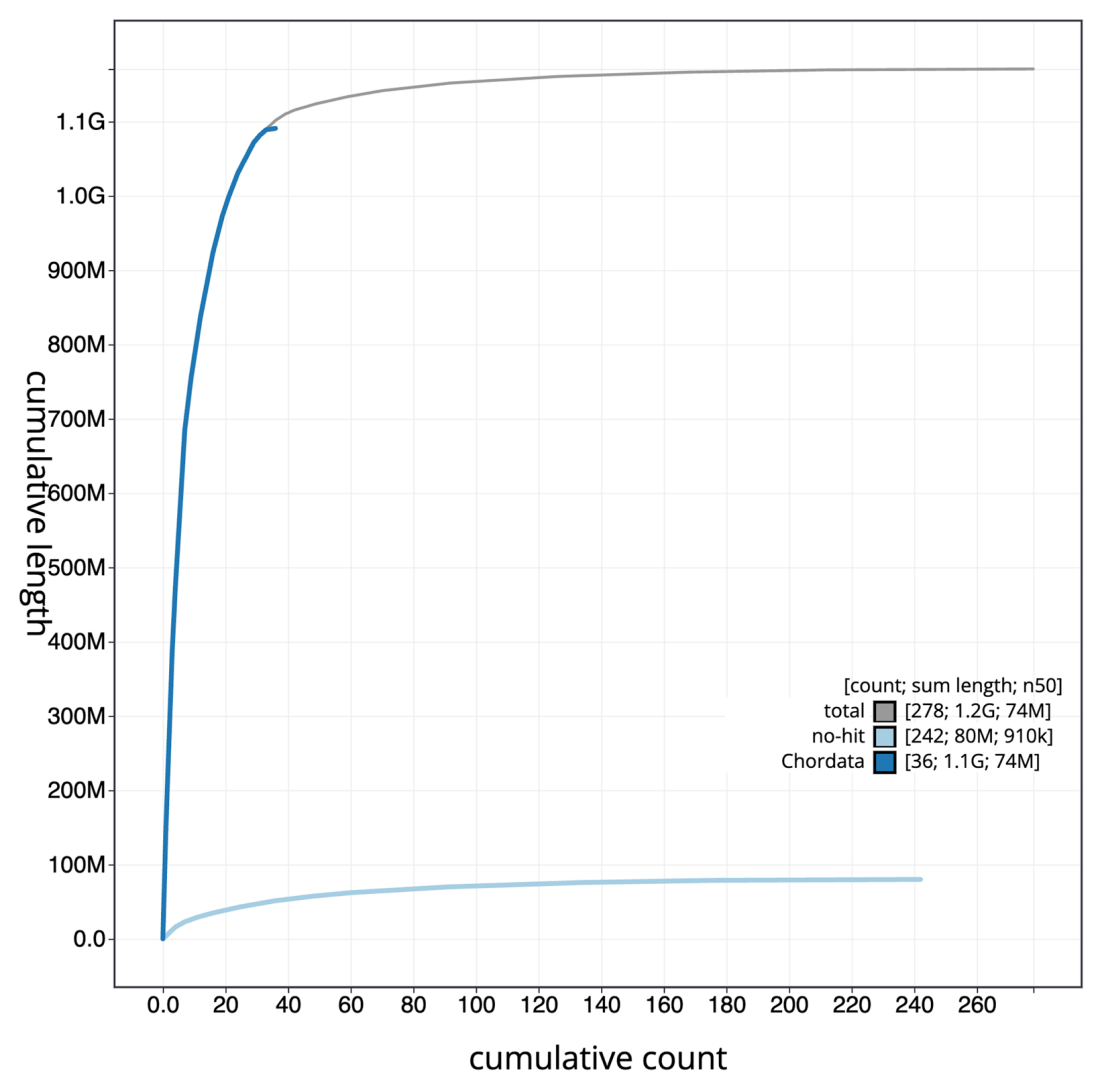
Genome assembly of *Cinclus cinclus* bCinCin1.1: BlobToolKit cumulative sequence plot. The grey line shows cumulative length for all scaffolds. Coloured lines show cumulative lengths of scaffolds assigned to each phylum using the buscogenes taxrule. An interactive version of this figure is available at https://blobtoolkit.genomehubs.org/view/GCA_963662255.1/dataset/GCA_963662255.1/cumulative.

Assembly errors were corrected by manual curation, including 103 missing joins or mis-joins and one haplotypic duplication. This reduced the scaffold number by 21.25% and increased the scaffold N50 by 4.02%. The final assembly has a total length of 1,170.80 Mb in 277 sequence scaffolds, with 658 gaps, and a scaffold N50 of 74.4 Mb ( Table 2). The snail plot in Figure 2 provides a summary of the assembly statistics, while the distribution of assembly scaffolds on GC proportion and coverage is shown in Figure 3. The cumulative assembly plot in Figure 4 shows curves for subsets of scaffolds assigned to different phyla.

**Table 2.** Genome assembly data for *Cinclus cinclus*, bCinCin1.1.

Genome assembly		
Assembly name	bCinCin1.1	
Assembly accession	GCA_963662255.1	
*Accession of alternate haplotype*	*GCA_963662415.1*	
Span (Mb)	1,170.80	
Number of contigs	936	
Number of scaffolds	277	
Longest scaffold (Mb)	152.57	
Assembly metrics *		*Benchmark*
Contig N50 length (Mb)	3.6	*≥ 1 Mb*
Scaffold N50 length (Mb)	74.4	*= chromosome N50*
Consensus quality (QV)	62.2	*≥ 40*
*k*-mer completeness	100.0%	*≥ 95%*
BUSCO **	C:96.5%[S:96.2%,D:0.3%], F:0.6%,M:2.9%,n:10,844	*S > 90%* *D < 5%*
Percentage of assembly mapped to chromosomes	93.88%	*≥ 90%*
Sex chromosomes	Z	*localised homologous pairs*
Organelles	Mitochondrial genome: 18.67 kb	*complete single alleles*

* Assembly metric benchmarks are adapted from Rhie *et al.* (2021) and the Earth BioGenome Project Report on Assembly Standards September 2024. ** BUSCO scores based on the passeriformes_odb10 BUSCO set using version 5.4.3. C = complete [S = single copy, D = duplicated], F = fragmented, M = missing, n = number of orthologues in comparison. A full set of BUSCO scores is available at https://blobtoolkit.genomehubs.org/view/Cinclus_cinclus/dataset/GCA_963662255.1/busco.

**Figure 5.**
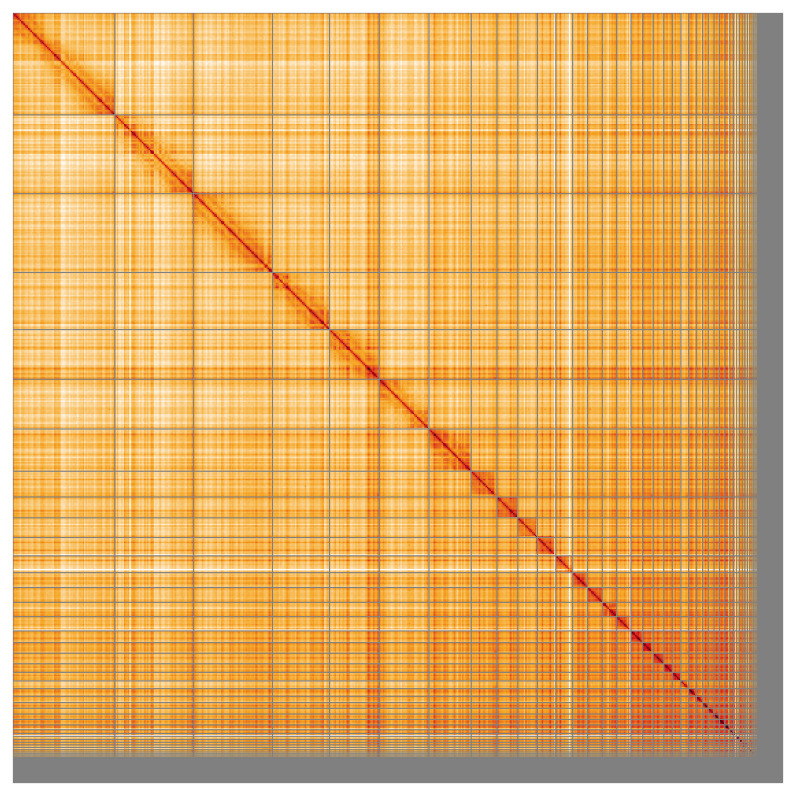
Genome assembly of *Cinclus cinclus* bCinCin1.1: Hi-C contact map of the bCinCin1.1 assembly, visualised using HiGlass. Chromosomes are shown in order of size from left to right and top to bottom. An interactive version of this figure may be viewed at https://genome-note-higlass.tol.sanger.ac.uk/l/?d=Oxsf2F9DQzWyNREabxewqA.

Most of the assembly sequence (93.88%) was assigned to 39 chromosomal-level scaffolds, representing 38 autosomes and the Z sex chromosome. Chromosome-scale scaffolds confirmed by the Hi-C data are named in order of size ( Figure 5; Table 3). The Z chromosome was identified based on synteny with *Taeniopygia guttata* (GCA_003957565.4). While not fully phased, the assembly deposited is of one haplotype. Contigs corresponding to the second haplotype have also been deposited. The mitochondrial genome was also assembled and can be found as a contig within the multifasta file of the genome submission.

**Table 3.** Chromosomal pseudomolecules in the genome assembly of *Cinclus cinclus*, bCinCin1.

INSDC accession	Name	Length (Mb)	GC%
OY759677.1	1	152.36	39.5
OY759678.1	2	117.95	40.0
OY759679.1	3	117.73	40.5
OY759681.1	4	74.41	40.5
OY759682.1	5	74.36	40.0
OY759683.1	6	62.87	41.0
OY759684.1	7	38.63	42.5
OY759685.1	8	30.98	42.0
OY759686.1	9	29.52	41.5
OY759687.1	10	27.25	43.5
OY759688.1	11	25.57	44.0
OY759689.1	12	22.06	44.5
OY759690.1	13	22.0	43.5
OY759691.1	14	21.63	46.0
OY759692.1	15	21.03	44.5
OY759693.1	16	17.5	45.5
OY759694.1	17	15.98	46.5
OY759695.1	18	15.8	46.5
OY759696.1	19	12.78	48.0
OY759697.1	20	12.67	47.5
OY759698.1	21	11.6	45.0
OY759699.1	22	11.48	47.0
OY759700.1	23	9.73	48.5
OY759701.1	24	8.45	51.5
OY759702.1	25	8.4	49.5
OY759703.1	26	8.3	51.0
OY759704.1	27	7.98	51.5
OY759705.1	28	7.3	52.0
OY759706.1	29	5.33	51.0
OY759707.1	30	4.62	54.0
OY759708.1	31	4.1	55.0
OY759709.1	32	3.79	53.0
OY759710.1	33	3.34	53.0
OY759711.1	34	1.58	54.0
OY759712.1	35	1.35	51.0
OY759713.1	36	1.3	50.5
OY759714.1	37	1.14	55.5
OY759715.1	38	0.74	55.0
OY759680.1	Z	85.38	41.0
OY759716.1	MT	0.02	46.0

The estimated Quality Value (QV) of the final assembly is 62.2 with *k*-mer completeness of 100.0%, and the assembly has a BUSCO v5.4.3 completeness of 96.5% (single = 96.2%, duplicated = 0.3%), using the passeriformes_odb10 reference set ( *n* = 10,844). The assembly achieves the Earth BioGenome Project standard of 6.C.62, exceeding the recommended minimum standard of 6.C.Q40. Other quality metrics are given in Table 2. 

Metadata for specimens, BOLD barcode results, spectra estimates, sequencing runs, contaminants and pre-curation assembly statistics are given at https://tolqc.cog.sanger.ac.uk/darwin/birds/Cinclus_cinclus/.

## Methods

### Sample acquisition

Blood samples were collected from four individual *Cinclus cinclus* in a long-term study population near Sedbergh, Cumbria, UK, under licence on 27 June 2021. Three nestlings of unknown sex were taken from the nest by hand at nine days old. A juvenile male was captured using a mist net. Blood was drawn via brachial venipuncture, transferred to Eppendorf tubes with capillary tubing, and stored in a –20°C freezer on dry ice. Stuart Sharp (Lancaster University) collected and identified the specimens.

The genome was sequenced from one of the nestlings (specimen ID SAN0001736, ToLID bCinCin1), later identified as a male. The juvenile male captured in the mist net (specimen ID SAN0001734, ToLID bCinCin2) was used for Hi-C sequencing. RNA sequencing was performed on another nestling of unknown sex (specimen ID SAN0001737, ToLID bCinCin4).

### Nucleic acid extraction

The workflow for high molecular weight (HMW) DNA extraction at the Wellcome Sanger Institute (WSI) Tree of Life Core Laboratory includes a sequence of core procedures: sample preparation and homogenisation, DNA extraction, fragmentation and purification. Detailed protocols are available on protocols.io ( Denton *et al.*, 2023b). The bCinCin1 blood sample was prepared for DNA extraction by weighing and dissecting it on dry ice ( Jay *et al.*, 2023), and homogenised using a PowerMasher II tissue disruptor ( Denton *et al.*, 2023a).

HMW DNA was extracted using the Automated MagAttract v2 protocol ( Oatley *et al.*, 2023a). DNA was sheared into an average fragment size of 12–20 kb in a Megaruptor 3 system ( Bates *et al.*, 2023). Sheared DNA was purified by solid-phase reversible immobilisation, using AMPure PB beads to eliminate shorter fragments and concentrate the DNA ( Oatley *et al.*, 2023b). The concentration of the sheared and purified DNA was assessed using a Nanodrop spectrophotometer and Qubit Fluorometer using the Qubit dsDNA High Sensitivity Assay kit. Fragment size distribution was evaluated by running the sample on the FemtoPulse system.

RNA was extracted from blood tissue of bCinCin4 in the Tree of Life Laboratory at the WSI using the RNA Extraction: Automated MagMax™ *mir*Vana protocol ( do Amaral *et al.*, 2023). The RNA concentration was assessed using a Nanodrop spectrophotometer and a Qubit Fluorometer using the Qubit RNA Broad-Range Assay kit. Analysis of the integrity of the RNA was done using the Agilent RNA 6000 Pico Kit and Eukaryotic Total RNA assay.

### Hi-C preparation

An aliquot of the bCinCin2 blood sample was processed at the WSI Scientific Operations core, using the Arima-HiC v2 kit. In brief, frozen tissue (stored at –80 °C) was fixed, and the DNA crosslinked using a TC buffer with 22% formaldehyde. After crosslinking, the tissue was homogenised using the Diagnocine Power Masher-II and BioMasher-II tubes and pestles. Following the kit manufacturer's instructions, crosslinked DNA was digested using a restriction enzyme master mix. The 5’-overhangs were then filled in and labelled with biotinylated nucleotides and proximally ligated. An overnight incubation was carried out for enzymes to digest remaining proteins and for crosslinks to reverse. A clean up was performed with SPRIselect beads prior to library preparation.

### Library preparation and sequencing

Library preparation and sequencing were performed at the WSI Scientific Operations core. Pacific Biosciences HiFi circular consensus DNA sequencing libraries were prepared using the PacBio Express Template Preparation Kit v2.0 (Pacific Biosciences, California, USA) as per the manufacturer's instructions. The kit includes the reagents required for removal of single-strand overhangs, DNA damage repair, end repair/A-tailing, adapter ligation, and nuclease treatment. Library preparation also included a library purification step using AMPure PB beads (Pacific Biosciences, California, USA) and size selection step to remove templates shorter than 3 kb using AMPure PB modified SPRI. DNA concentration was quantified using the Qubit Fluorometer v2.0 and Qubit HS Assay Kit and the final library fragment size analysis was carried out using the Agilent Femto Pulse Automated Pulsed Field CE Instrument and gDNA 165kb gDNA and 55kb BAC analysis kit. Samples were sequenced using the Sequel IIe system (Pacific Biosciences, California, USA). The concentration of the library loaded onto the Sequel IIe was between 40–135 pM. The SMRT link software, a PacBio web-based end-to-end workflow manager, was used to set-up and monitor the run, as well as perform primary and secondary analysis of the data upon completion.

Poly(A) RNA-Seq libraries were constructed using the NEB Ultra II RNA Library Prep kit, following the manufacturer’s instructions. RNA sequencing was performed on Illumina NovaSeq 6000 instrument.

For Hi-C library preparation, DNA was fragmented to a size of 400 to 600 bp using a Covaris E220 sonicator. The DNA was then enriched, barcoded, and amplified using the NEBNext Ultra II DNA Library Prep Kit following manufacturers’ instructions. The Hi-C sequencing was performed using paired-end sequencing with a read length of 150 bp on an Illumina NovaSeq 6000 instrument.

### Genome assembly, curation and evaluation

***Assembly***

The HiFi reads were first assembled using Hifiasm ( Cheng *et al.*, 2021) with the --primary option. Haplotypic duplications were identified and removed using purge_dups ( Guan *et al.*, 2020). The Hi-C reads were mapped to the primary contigs using bwa-mem2 ( Vasimuddin *et al.*, 2019). The contigs were further scaffolded using the provided Hi-C data ( Rao *et al.*, 2014) in YaHS ( Zhou *et al.*, 2023) using the --break option. The scaffolded assemblies were evaluated using Gfastats ( Formenti *et al.*, 2022), BUSCO ( Manni *et al.*, 2021) and MERQURY.FK ( Rhie *et al.*, 2020).

The mitochondrial genome was assembled using MitoHiFi ( Uliano-Silva *et al.*, 2023), which runs MitoFinder ( Allio *et al.*, 2020) and uses these annotations to select the final mitochondrial contig and to ensure the general quality of the sequence.

***Assembly curation***

The assembly was decontaminated using the Assembly Screen for Cobionts and Contaminants (ASCC) pipeline (article in preparation). Flat files and maps used in curation were generated in TreeVal ( Pointon *et al.*, 2023). Manual curation was primarily conducted using PretextView ( Harry, 2022), with additional insights provided by JBrowse2 ( Diesh *et al.*, 2023) and HiGlass ( Kerpedjiev *et al.*, 2018). Scaffolds were visually inspected and corrected as described by Howe *et al*. (2021). Any identified contamination, missed joins, and mis-joins were corrected, and duplicate sequences were tagged and removed. The Z chromosome was assigned by synteny analysis. The curation process is documented at https://gitlab.com/wtsi-grit/rapid-curation (article in preparation).

***Evaluation of the final assembly***

The final assembly was post-processed and evaluated using the three Nextflow ( Di Tommaso *et al.*, 2017) DSL2 pipelines: sanger-tol/readmapping ( Surana *et al.*, 2023a), sanger-tol/genomenote ( Surana *et al.*, 2023b), and sanger-tol/blobtoolkit ( Muffato *et al.*, 2024). The readmapping pipeline aligns the Hi-C reads using bwa-mem2 ( Vasimuddin *et al.*, 2019) and combines the alignment files with SAMtools ( Danecek *et al.*, 2021). The genomenote pipeline converts the Hi-C alignments into a contact map using BEDTools ( Quinlan & Hall, 2010) and the Cooler tool suite ( Abdennur & Mirny, 2020). The contact map is visualised in HiGlass ( Kerpedjiev *et al.*, 2018). This pipeline also generates assembly statistics using the NCBI datasets report ( Sayers *et al.*, 2024), computes *k*-mer completeness and QV consensus quality values with FastK and MERQURY.FK, and runs BUSCO ( Manni *et al.*, 2021) to assess completeness.

The blobtoolkit pipeline is a Nextflow port of the previous Snakemake Blobtoolkit pipeline ( Challis *et al.*, 2020). It aligns the PacBio reads in SAMtools and minimap2 ( Li, 2018) and generates coverage tracks for regions of fixed size. In parallel, it queries the GoaT database ( Challis *et al.*, 2023) to identify all matching BUSCO lineages to run BUSCO ( Manni *et al.*, 2021). For the three domain-level BUSCO lineages, the pipeline aligns the BUSCO genes to the UniProt Reference Proteomes database ( Bateman *et al.*, 2023) with DIAMOND ( Buchfink *et al.*, 2021) blastp. The genome is also split into chunks according to the density of the BUSCO genes from the closest taxonomic lineage, and each chunk is aligned to the UniProt Reference Proteomes database with DIAMOND blastx. Genome sequences without a hit are chunked with seqtk and aligned to the NT database with blastn ( Altschul *et al.*, 1990). The blobtools suite combines all these outputs into a blobdir for visualisation.

The genome assembly and evaluation pipelines were developed using nf-core tooling ( Ewels *et al.*, 2020) and MultiQC ( Ewels *et al.*, 2016), relying on the Conda package manager, the Bioconda initiative ( Grüning *et al.*, 2018), the Biocontainers infrastructure ( da Veiga Leprevost *et al.*, 2017), as well as the Docker ( Merkel, 2014) and Singularity ( Kurtzer *et al.*, 2017) containerisation solutions.

Table 4 contains a list of relevant software tool versions and sources.

**Table 4.** Software tools: versions and sources.

Software tool	Version	Source
BEDTools	2.30.0	https://github.com/arq5x/bedtools2
BLAST	2.14.0	ftp://ftp.ncbi.nlm.nih.gov/blast/executables/blast+/
BlobToolKit	4.3.7	https://github.com/blobtoolkit/blobtoolkit
BUSCO	5.4.3 and 5.5.0	https://gitlab.com/ezlab/busco
bwa-mem2	2.2.1	https://github.com/bwa-mem2/bwa-mem2
Cooler	0.8.11	https://github.com/open2c/cooler
DIAMOND	2.1.8	https://github.com/bbuchfink/diamond
fasta_windows	0.2.4	https://github.com/tolkit/fasta_windows
FastK	427104ea91c78c3b8b8b49f1a7d6bbeaa869ba1c	https://github.com/thegenemyers/FASTK
Gfastats	1.3.6	https://github.com/vgl-hub/gfastats
GoaT CLI	0.2.5	https://github.com/genomehubs/goat-cli
Hifiasm	0.16.1-r375	https://github.com/chhylp123/hifiasm
HiGlass	44086069ee7d4d3f6f3f0012569789ec138f42b84 aa44357826c0b6753eb28de	https://github.com/higlass/higlass
Merqury.FK	d00d98157618f4e8d1a9190026b19b471055b22e	https://github.com/thegenemyers/MERQURY.FK
MitoHiFi	3	https://github.com/marcelauliano/MitoHiFi
MultiQC	1.14, 1.17, and 1.18	https://github.com/MultiQC/MultiQC
NCBI Datasets	15.12.0	https://github.com/ncbi/datasets
Nextflow	23.04.0-5857	https://github.com/nextflow-io/nextflow
PretextView	0.2	https://github.com/sanger-tol/PretextView
purge_dups	1.2.5	https://github.com/dfguan/purge_dups
samtools	1.16.1, 1.17, and 1.18	https://github.com/samtools/samtools
sanger-tol/ascc	-	https://github.com/sanger-tol/ascc
sanger-tol/genomenote	1.1.1	https://github.com/sanger-tol/genomenote
sanger-tol/readmapping	1.2.1	https://github.com/sanger-tol/readmapping
Seqtk	1.3	https://github.com/lh3/seqtk
Singularity	3.9.0	https://github.com/sylabs/singularity
TreeVal	1.0.0	https://github.com/sanger-tol/treeval
YaHS	1.2a.2	https://github.com/c-zhou/yahs

### Wellcome Sanger Institute – Legal and Governance

The materials that have contributed to this genome note have been supplied by a Darwin Tree of Life Partner. The submission of materials by a Darwin Tree of Life Partner is subject to the **‘Darwin Tree of Life Project Sampling Code of Practice’**, which can be found in full on the Darwin Tree of Life website here. By agreeing with and signing up to the Sampling Code of Practice, the Darwin Tree of Life Partner agrees they will meet the legal and ethical requirements and standards set out within this document in respect of all samples acquired for, and supplied to, the Darwin Tree of Life Project. 

Further, the Wellcome Sanger Institute employs a process whereby due diligence is carried out proportionate to the nature of the materials themselves, and the circumstances under which they have been/are to be collected and provided for use. The purpose of this is to address and mitigate any potential legal and/or ethical implications of receipt and use of the materials as part of the research project, and to ensure that in doing so we align with best practice wherever possible. The overarching areas of consideration are:

•   Ethical review of provenance and sourcing of the material

•   Legality of collection, transfer and use (national and international) 

Each transfer of samples is further undertaken according to a Research Collaboration Agreement or Material Transfer Agreement entered into by the Darwin Tree of Life Partner, Genome Research Limited (operating as the Wellcome Sanger Institute), and in some circumstances other Darwin Tree of Life collaborators.

## Data Availability

European Nucleotide Archive: *Cinclus cinclus* (white-throated dipper). Accession number PRJEB61999; https://identifiers.org/ena.embl/PRJEB61999 ( Wellcome Sanger Institute, 2023). The genome sequence is released openly for reuse. The *Cinclus cinclus* genome sequencing initiative is part of the Darwin Tree of Life (DToL) project. All raw sequence data and the assembly have been deposited in INSDC databases. The genome will be annotated using available RNA-Seq data and presented through the Ensembl pipeline at the European Bioinformatics Institute. Raw data and assembly accession identifiers are reported in Table 1 and Table 2.

## Author information

Members of the Wellcome Sanger Institute Tree of Life Management, Samples and Laboratory team are listed here: https://doi.org/10.5281/zenodo.12162482.

Members of Wellcome Sanger Institute Scientific Operations: Sequencing Operations are listed here: https://doi.org/10.5281/zenodo.12165051.

Members of the Wellcome Sanger Institute Tree of Life Core Informatics team are listed here: https://doi.org/10.5281/zenodo.12160324.

Members of the Tree of Life Core Informatics collective are listed here: https://doi.org/10.5281/zenodo.12205391.

Members of the Darwin Tree of Life Consortium are listed here: https://doi.org/10.5281/zenodo.4783558.
