# Supplementary material for: The genome sequence of the white-throated dipper, Cinclus cinclus (Linnaeus, 1758)
Source: Wellcome Open Res. 2024 Nov 5;9:645. [Version 1] doi: 10.12688/wellcomeopenres.23291.1 (PMC11747308; doi:10.12688/wellcomeopenres.23291.1)
Supplement: Peer review history

# Reviewer report: Reviewer response for version 1

**DOI:** 10.21956/wellcomeopenres.25673.r112516

**Published:** 2025-01-21

**Authors:** Alexandrex Alexandre Aleixo

**Affiliations:** 1 Instituto Tecnológico Vale - Desenvolvimento Sustentável (ITV-DS), Belacm, Brazil

This is an important contribution to genomics and ornithology. I have a few comments below which should be addressed by the authors in a second version of their manuscript:

 1) Are the three nestlings taken from the nest siblings? Do you know whether the specimen caught in the net was related to the nestlings? Since sequencing was done from two individuals, any information about their relationships is relevant and should be reported. 

 2) What does MP mean un table 3 (Chromosomal pseudomolecules)? Please clarify in a legend.

Are sufficient details of methods and materials provided to allow replication by others?

Partly

Is the rationale for creating the dataset(s) clearly described?

Yes

Are the datasets clearly presented in a useable and accessible format?

Yes

Are the protocols appropriate and is the work technically sound?

Yes

Reviewer Expertise:

Genomics and Zoology; population genomics and phylogeography.

I confirm that I have read this submission and believe that I have an appropriate level of expertise to confirm that it is of an acceptable scientific standard.

# Reviewer report: Reviewer response for version 1

**DOI:** 10.21956/wellcomeopenres.25673.r113370

**Published:** 2025-01-20

**Authors:** Bertrand Bed'Hom

**Affiliations:** 1 Institut de Systematique Evolution Biodiversite, Paris, Île-de-France, France

This article presents a reference genome for the white-throated dipper ( *Cinclus cinclus*). The protocol is well described and follows the standard practices of the Sanger Institute for the Darwin Tree of Life. The quality of the genome assembly seems very good. The scaffold number is compatible with the expected chromosome number, as the closely related *Cinclus pallasii* species has 2N=78 chromosomes (Li et al., 1994).

 I have only two minor comments :

 - "for waterproofing" is a bit  teleological

 - "41-fold coverage" : it's 41-fold depth here. Coverage should be kept for a proportion of sequence length (as in GC-coverage).

Are sufficient details of methods and materials provided to allow replication by others?

Yes

Is the rationale for creating the dataset(s) clearly described?

Yes

Are the datasets clearly presented in a useable and accessible format?

Yes

Are the protocols appropriate and is the work technically sound?

Yes

Reviewer Expertise:

Genomics, Cytogenetics, Evolution

I confirm that I have read this submission and believe that I have an appropriate level of expertise to confirm that it is of an acceptable scientific standard.

## References

[1] Li Q, Zhang H, Wang Y, Bian X. Studies on the karyotypes of birds XII. The 12 species of Passeriformes. Zoological Research 15:33-37, 1994.

# Reviewer report: Reviewer response for version 1

**DOI:** 10.21956/wellcomeopenres.25673.r113369

**Published:** 2024-12-24

**Authors:** Annabel Whibley

**Affiliations:** 1 The University of Auckland School of Biological Sciences, Auckland, Auckland, New Zealand; 2 Grapevine Improvement, Bragato Research Institute, Lincoln, New Zealand

Sharp and colleagues present a high quality genome assembly of the passerine bird the white-throated dipper ( *Cinclus cinclus). *The assembly is generated from PacBio and HiC datasets and annotations are supported by RNA sequence data. All metadata pertaining to sample collection, lab and bioinformatics protocols are clearly recorded, and both intermediate and final data outputs are publicly available and easily findable. The Data Note is well written and the high quality genomics resources produced by this work will be of value to the scientific community.

Are sufficient details of methods and materials provided to allow replication by others?

Yes

Is the rationale for creating the dataset(s) clearly described?

Yes

Are the datasets clearly presented in a useable and accessible format?

Yes

Are the protocols appropriate and is the work technically sound?

Yes

Reviewer Expertise:

Bioinformatics, Genomics

I confirm that I have read this submission and believe that I have an appropriate level of expertise to confirm that it is of an acceptable scientific standard.
